# Higher atherogenic index of plasma is associated with increased major depressive disorder: insights from a nationally representative study

**DOI:** 10.3389/fpsyt.2024.1441119

**Published:** 2024-10-10

**Authors:** Shiyi Tao, Lintong Yu, Jun Li, Xuanchun Huang, Tiantian Xue, Deshuang Yang, Yuqing Tan

**Affiliations:** ^1^ Department of Cardiology, Guang'anmen Hospital, China Academy of Chinese Medical Sciences, Beijing, China; ^2^ Graduate School, Beijing University of Chinese Medicine, Beijing, China; ^3^ Department of Integrative Cardiology, China-Japan Friendship Hospital, Beijing, China

**Keywords:** atherogenic index of plasma, major depressive disorder, dyslipidemia, PHQ-9, NHANES

## Abstract

**Background:**

Emerging studies reveal a shared pathophysiological underpinning for metabolic problems and mental illnesses. The present study aimed to determine the association between atherogenic index of plasma (AIP) and the incidence of major depressive disorder (MDD).

**Methods:**

7,951 subjects of US adults were collected from the National Health and Nutrition Examination Survey (NHANES) 2005-2018. MDD was evaluated through the Patient Health Questionnaire (PHQ-9). Multivariate logistic regression, sensitivity analysis, and spline smoothing plot method were used to identify the relationship between AIP and MDD. The cut-off point was calculated using recursive partitioning analysis when segmenting effects emerged. The area under the receiver operating characteristic (ROC) curve (AUC) and Hosmer-Lemeshow test were conducted to evaluate the performance of AIP in identifying MDD. Subgroup analyses and interaction tests were used to explore whether the association was stable in different populations.

**Results:**

A positive correlation between AIP and PHQ-9 score and MDD was both observed in 7,951 subjects included in the study, with a significant threshold of -0.42 determined using recursive partitioning analysis. In the fully adjusted model, a positive association between AIP and PHQ-9 score and MDD was observed (β=0.46, 95% CI 0.14~0.78; OR=1.42, 95% CI 1.04~1.93). Individuals in the highest AIP quartile had a 0.39-unit higher PHQ-9 score (β=0.39, 95% CI 0.12~0.66) and a significantly 33% greater risk of MDD than those in the lowest AIP quartile (OR=1.33, 95% CI 1.02~1.73). Spline smoothing plot analysis further confirmed the positive and non-linear association between AIP and PHQ-9 and MDD. ROC analysis (AUC=0.771) and the Hosmer-Lemeshow test (χ^2^ = 14.239, *P*=0.076) suggested an excellent performance and goodness-of-fit of the relatively optimal model. DCA and CIC analysis also revealed a favorable overall net benefit and clinical impact of the model. Subgroup analyses and interaction tests revealed that the association between AIP and PHQ-9 score and MDD remained consistent across different subgroups and was not modified by other covariates, and this positive correlation was more pronounced in those with diabetes or hypertension.

**Conclusion:**

An elevated AIP is linked to a higher chance of MDD, especially in those with diabetes or hypertension. Resolving dyslipidemia and managing comorbidities may help reduce the likelihood of developing MDD.

## 1 Introduction

Depressive disorder is a widespread, severe, and possibly lethal mental condition that also harms physical health and imposes a significant cost on public health (1). Around 300 million individuals worldwide live in the shadow of depressive disorder, and the Global cases of major depressive disorder (MDD) increased by 27.6% in the wake of the novel coronavirus pneumonia pandemic, posing a significant challenge to mental health systems and services (2). Depressive disorders are expected to surpass cardiovascular diseases and tumors as the first global burden of disease in 2030 (3). Currently, the management of depressive disorder comprises a variety of ways such as pharmacotherapy, psychotherapy, electroconvulsive treatment, and integrative therapy (4). Although antidepressants are widely available and relatively effective, they often produce adverse reactions and take weeks before exhibiting effectiveness, thus limiting their use in certain populations, including those with acute illnesses, adolescents, and pregnant women (5, 6). Accordingly, predicting and preventing depression is crucial, and an easily accessible and reliable predictor to assess the occurrence and development of depressive disorders is of paramount importance in clinical settings.

The pathogenesis of depressive disorders is complicated and can be influenced by numerous factors, including genetic, psychological, environmental, and biological factors. The links between depression and oxidative stress, pro-inflammatory response, as well as the widely studied relationships between depression and glucose tolerance disorders were well developed (7, 8). Recently, lipid metabolism has been shown to play an important role in the progression of psychiatric disorders such as depressive disorder (9). According to earlier studies, individuals who suffered from depressive disorder frequently had certain alterations in their lipid profiles, including raised levels of total cholesterol (TC), elevated levels of low-density lipoprotein cholesterol (LDL-C) and triglyceride (TG), and lower levels of high-density lipoprotein cholesterol (HDL-C) (10, 11). The atherogenic index of plasma (AIP), an important biomarker for predicting cardiovascular diseases and depressive symptoms, is mathematically defined as the logarithmic ratio of TG to HDL-C (12). The relationship between cardiovascular events and mental health has been well established (13–15). As studies increasingly suggests that AIP is associated with the incident cardiovascular events, it becomes imperative to identify whether AIP may also reveal complex interactions between cardiovascular diseases and depression. Previous studies have confirmed the relationship between AIP and cardiovascular diseases as well as the association of cardiovascular diseases with depression, respectively (16, 17). Nevertheless, there is still a lack of evidence on the potential direct association between AIP and MDD. Most importantly, determining new and dependable biomarkers that may help predict MDD would contribute to early detection, risk stratification and timely intervention, ultimately improving patient outcomes.

Therefore, this study aims to examine the relationship between AIP and depressive disorder using the continuous cross-sectional data from the NHANES database with national representation and complex multi-stage sampling. Our findings may provide convincing evidence on the involvement of lipid metabolism in MDD and serve as an informative guide for revealing the potential mechanism and correlation between AIP and MDD.

## 2 Materials and methods

### 2.1 Study population

This study was conducted with data sourced from the National Health and Nutrition Examination Survey (NHANES) carried out from 2005 to 2018. The NHANES database is a national nutrition and health survey of the U.S. population conducted annually since 1999 by the National Center for Health Statistics (NCHS) of the Centers for Disease Control and Prevention (CDC). The survey utilized household questionnaires, telephone interviews, and examinations conducted by medical professionals and trained personnel to collect data and to create a representative sample of the U.S. population through complex multistage, stratified, clustered probability sample. The survey protocol was formally approved by the CDC Institutional Review Board, and all participants gave voluntary informed consent.

**Figure 1:**
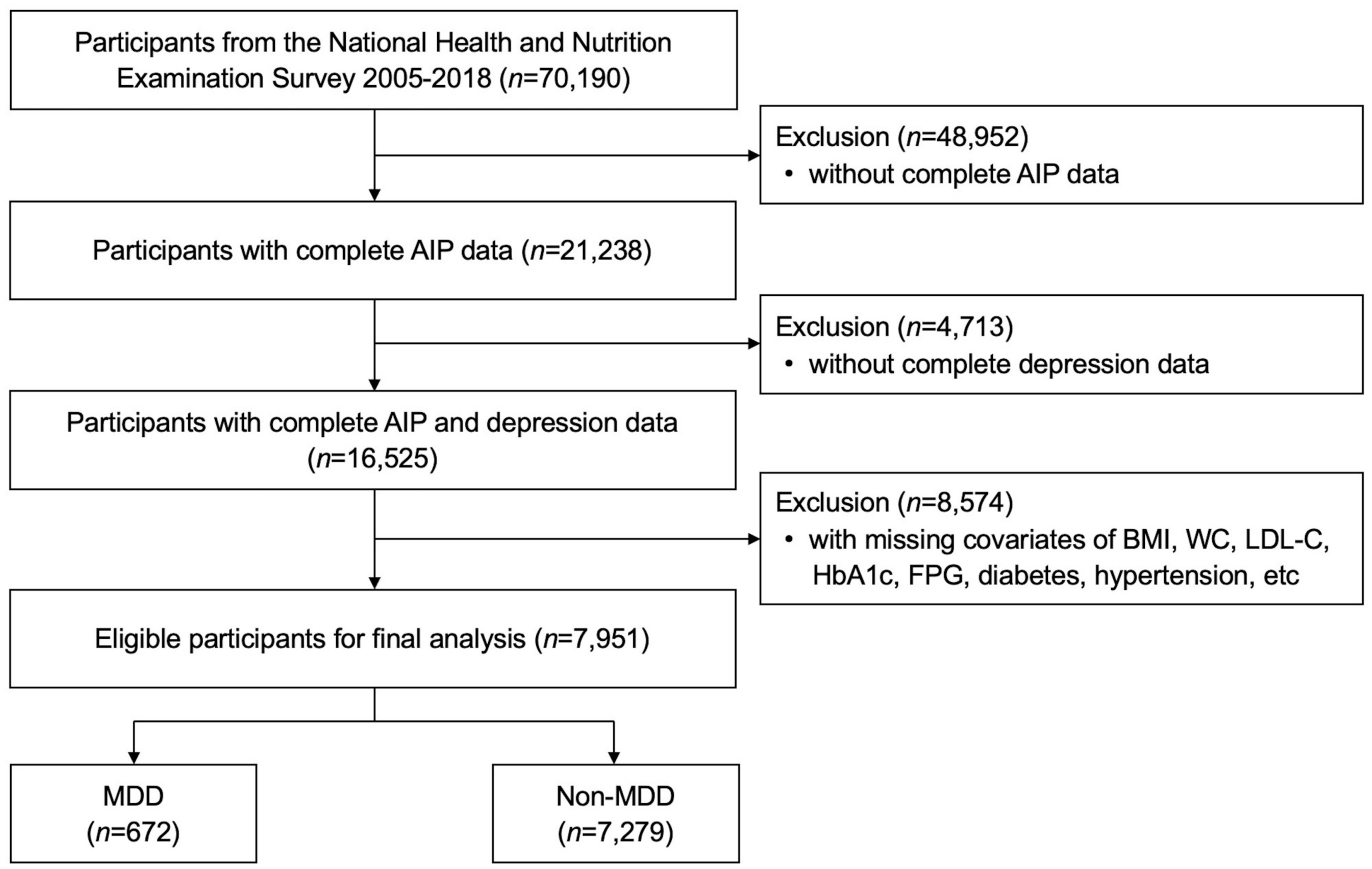
Flow diagram of participant selection.

As shown in **Figure 1**, seven cycles of dataset with full information were used for analysis 2005-2018. Among the 70,190 subjects in the NHANES over the period 2005-2018, a total of 7,951 US adults were enrolled in the present study finally after exclusion of patients with no complete AIP data (n=48,952) and MDD information (n=4,713), and with missing covariates (n=8,574).

### 2.2 Major depressive disorder

Depressive disorder was assessed using the Patient Health Questionnaire (PHQ-9), a self-report depression screening instrument from the Diagnostic and Statistical Manual of Mental Disorders, Fourth Edition (18). It is a dependable and reliable tool of depression severity based on nine items reflecting depressive disorder that capture the signs and symptoms of depression experienced over the past two weeks. For each symptom question, points ranging from 0 to 3, are associated with the response categories “not at all”, “several days”, “more than half the days”, and “nearly every day”. Total scores range from 0 to 27, with a score greater than or equal to 10 representing MDD (19).

### 2.3 Atherogenic index of plasma

AIP is derived from two vital lipid indicators, including TG and HDL-C. Rigorous laboratory tests for relevant indicators were conducted in accordance with standardized sampling protocols to assure the validity and comparability of data. Blood samples are typically collected on survey vehicles or at designated sampling sites, followed by processing and testing in the laboratory. The computational formula for AIP was presented as follows: AIP=Lg [TG (mmol/L)/HDL-C (mmol/L)] (12).

### 2.4 Covariates

The covariates were selected based on previous studies and clinical practice that demonstrated a potential association with AIP levels and MDD prevalence. Standardized questionnaires were utilized to gather information on various factors, including demographic, medical history, and laboratory data. Demographic data comprised age, gender, race, education level, marital status, family income-to-poverty ratio (FIPR), body mass index (BMI), and waist circumference (WC). Medical history information included PHQ-9 score, cigarette use, diabetes, hypertension, hyperlipidemia, cardiovascular disease (CVD), and chronic kidney disease (CKD). Laboratory data encompassed, white blood cell (WBC), segmented neutrophil (Neu), lymphocyte (Lym), platelet (PLT), hemoglobin (Hb), alanine aminotransferase (ALT), aspartate aminotransferase (AST), alkaline phosphatase (ALP), gamma glutamyl transferase (GGT), serum creatinine (Scr), uric acid (UA), TC, TG, LDL-C, HDL-C, fasting plasma glucose (FPG), glycated hemoglobin A1c (HbA1c), iron, phosphorus, potassium, sodium, and calcium. The demographic data and medical history information were collected through interviews. Race was categorized into four groups: Mexican American, Non-Hispanic White, Non-Hispanic Black, and other races. Education was stratified into three classes: less than high school, high school or equivalent, and college graduate or above. Marital status was organized into three groups: never married, married or living with partner, and others (widowed, divorced, and separated). Smoking at least 100 cigarettes in entire life was defined as a smoker. Detailed information on the specimen collection, processing, quality assurance, and monitoring are described in the section of biospecimen program in NHANES.

### 2.5 Statistical analysis

Continuous variables were expressed as mean ± standard deviation (SD) or median [interquartile range (IQR)], and the *t*-test or the Mann-Whitney *U* test was selected for hypothesis testing for those with normal and skewed distributions, respectively. Categorical variables were summarized as percentage-based figures and compared by the Chi-Square test. Multivariate linear and logistic regression model was used to explore the relationship between AIP and PHQ-9 score and MDD, respectively. AIP was treated as a continuous variable in the present analysis, and subjects were grouped based on the AIP quartiles for further analysis. Moreover, we established three regression models by adjusting different indicators to control for confounding biases. In addition to the Model 1 without any adjustments for confounders, two other models were fitted. In Model 2, gender, age, race, education level, marital status, and FIPR were modified. Model 3 was a fully adjusted model that took gender, age, race, education level, marital status, FIPR, BMI, diabetes, hypertension, hyperlipidemia, CVD, CKD, and FPG into account. Indicators that were found to be associated with MDD in previous studies or were statistically different at baseline characteristics were also considered to construct the model. The performance of AIP in identifying MDD was assessed using the area under the receiver operating characteristic (ROC) curve (AUC). Meanwhile, we assessed the goodness-of-fit and reliability of the relatively optimal models using calibration curve and Hosmer-Lemeshow test, with *P*>0.05 regarded as an acceptable model. The decision curve analysis (DCA) and clinical impact curve (CIC) were employed to assess the predictive accuracy and clinical value of the model. Subgroup analyses and interaction tests were used to confirm the stability of the association between AIP and PHQ-9 score and MDD across different subgroups.

Statistical analyses were performed using IBM-SPSS (version 26.0, Chicago, IL, USA) and R (version 4.1.2, Vienna, Austria). A two-sided *P*<0.05 was considered statistically significant.

## 3 Results

### 3.1 Baseline characteristics

A total of 7,951 subjects with an average age of 60 years were enrolled in the present study, of whom 49.23% were male. The mean AIP levels was -0.05 (-0.26-0.16) for all participants, with the prevalence of MDD was 8.45% overall.

The baseline characteristics of the study subjects according to the PHQ-9 score as a column stratification variable are shown in **Table 1**. MDD was defined as a PHQ-9 score greater than or equal to 10. Individuals with higher PHQ-9 scores were more likely to be female, younger, less than high school/high school or equivalent, never married, and low income. Depressive disorder was also significantly different in smoking, diabetes, hypertension, hyperlipidemia, CVD, and CKD (*P*<0.001). Meanwhile, subjects in the MDD group tended to have elevated AIP, BMI, WC, WBC, Neu, Lym, PLT, ALP, GGT, TG, FPG, HbA1c, serum phosphorus, and lower Hb, AST, UA, HDL-C, serum iron, and sodium levels (*P*<0.05). No statistically significant differences were recorded between the two group in race, ALT, Scr, TC, LDL-C, serum potassium, and calcium (*P*>0.05).

**Table 1:** Baseline characteristics of study subjects.

Variables	All	MDD	Non-MDD	*P* value
N	7,951	672	7,279	
Demographics				
Age (years)	60.00 (50.00-69.00)	58.00 (49.00-65.25)	60.00 (50.00-70.00)	<0.001
Gender				<0.001
Male (n, %)	3914 (49.23)	251 (37.35)	3663 (50.32)	
Female (n, %)	4037 (50.77)	421 (62.65)	3616 (49.68)	
Race (n, %)				0.516
Mexican American	1003 (12.61)	97 (14.43)	906 (12.45)	
Non-Hispanic White	3876 (48.75)	323 (48.07)	3553 (48.81)	
Non-Hispanic Black	1578 (19.85)	131 (19.49)	1447 (19.88)	
Others	1494 (18.79)	121 (18.01)	1373 (18.86)	
Education levels				<0.001
Less than high school	1842 (23.17)	237 (35.27)	1605 (22.05)	
High school or equivalent	1832 (23.04)	166 (24.70)	1666 (22.89)	
College or above	4277 (53.79)	269 (40.03)	4008 (55.06)	
Marital status				<0.001
Never married	601 (7.56)	80 (11.90)	521 (7.16)	
Married or living with partner	5081 (63.90)	301 (44.79)	4780 (65.67)	
Others	2269 (28.54)	291 (43.30)	1978 (27.17)	
FIPR	2.43 (1.27-4.54)	1.27 (0.79-2.45)	2.57 (1.35-4.69)	<0.001
BMI (kg/m^2^)	28.35 (24.90-32.80)	30.26 (25.80-35.09)	28.2 (24.87-32.50)	<0.001
WC (cm)	100.30 (91.00-110.80)	104.25 (94.00-115.12)	100.00 (90.80-110.30)	<0.001
Medical history (n, %)				
PHQ-9 score	2.00 (0.00-4.00)	13.00 (11.00-16.25)	1.00 (0.00-3.00)	<0.001
Smoker				<0.001
Yes	3893 (48.96)	413 (61.46)	3480 (47.81)	
No	4058 (51.04)	259 (38.54)	3799 (52.19)	
Diabetes				<0.001
Yes	1488 (18.71)	187 (27.83)	1301 (17.87)	
No	6463 (81.29)	485 (72.17)	5978 (82.13)	
Hypertension				<0.001
Yes	3884 (48.85)	404 (60.12)	3480 (47.81)	
No	4067 (51.15)	268 (39.88)	3799 (52.19)	
Hyperlipidemia				<0.001
Yes	3760 (47.29)	368 (54.76)	3392 (46.60)	
No	4191 (52.71)	304 (45.24)	3887 (53.40)	
CVD				<0.001
Yes	2219 (27.91)	388 (57.74)	1831 (25.15)	
No	5732 (72.09)	284 (42.26)	5448 (74.85)	
CKD				<0.001
Yes	307 (3.86)	53 (7.89)	254 (3.49)	
No	7644 (96.14)	619 (92.11)	7025 (96.51)	
Laboratory results				
WBC (1000 cells/uL)	6.40 (5.40-7.80)	7.10 (5.70-8.60)	6.40 (5.30-7.70)	<0.001
Neu (1000 cells/uL)	3.70 (2.90-4.70)	4.20 (3.10-5.30)	3.60 (2.90-4.70)	<0.001
Lym (1000 cells/uL)	1.90 (1.50-2.30)	1.95 (1.60-2.52)	1.90 (1.50-2.30)	0.008
PLT (1000 cells/uL)	232.00 (194.00-276.00)	241.50 (202.00-288.00)	231.00 (194.00-274.00)	<0.001
Hb (g/dL)	14.20 (13.20-15.10)	13.80 (12.88-15.00)	14.20 (13.20-15.10)	<0.001
ALT (U/L)	21.00 (16.00-28.00)	20.00 (16.00-28.00)	21.00 (16.00-28.00)	0.241
AST (U/L)	23.00 (19.00-27.00)	22.00 (18.00-28.00)	23.00 (19.00-27.00)	0.009
ALP (U/L)	68.00 (56.00-84.00)	74.00 (61.00-90.25)	68.00 (56.00-83.00)	<0.001
GGT (IU/L)	21.00 (15.00-32.00)	23.00 (17.00-38.25)	21.00 (15.00-31.00)	<0.001
Scr (*μ*mol/L)	76.91 (64.53-90.17)	73.37 (61.88-89.28)	77.79 (64.53-91.05)	0.381
UA (*μ*mol/L)	327.10 (273.60-386.60)	312.25 (261.70-374.70)	327.10 (273.60-386.60)	<0.001
TC (mmol/L)	4.99 (4.29-5.72)	4.94 (4.22-5.69)	4.99 (4.29-5.72)	0.937
TG (mmol/L)	1.20 (0.86-1.73)	1.35 (0.95-1.94)	1.18 (0.85-1.71)	<0.001
LDL-C (mmol/L)	2.92 (2.33-3.57)	2.90 (2.30-3.57)	2.92 (2.35-3.57)	0.642
HDL-C (mmol/L)	1.34 (1.11-1.66)	1.29 (1.06-1.58)	1.37 (1.11-1.66)	<0.001
FPG (mmol/L)	5.77 (5.33-6.44)	5.88 (5.38-6.85)	5.77 (5.33-6.44)	<0.001
HbA1c (%)	5.70 (5.40-6.10)	5.70 (5.40-6.30)	5.60 (5.40-6.00)	<0.001
Iron (*μ*mol/L)	15.20 (11.80-19.30)	14.10 (10.40-18.40)	15.40 (11.80-19.30)	<0.001
Phosphorus (mmol/L)	1.16 (1.07-1.29)	1.20 (1.07-1.32)	1.16 (1.07-1.29)	<0.001
Potassium (mmol/L)	4.00 (3.80-4.30)	4.00 (3.80-4.30)	4.00 (3.80-4.30)	0.504
Sodium (mmol/L)	140.00 (138.00-141.00)	139.00 (138.00-141.00)	140.00 (138.00-141.00)	0.005
Calcium (mmol/L)	2.33 (2.27-2.40)	2.33 (2.27-2.40)	2.33 (2.27-2.40)	0.160
AIP	-0.05 (-0.26-0.16)	0.02 (-0.20-0.23)	-0.06 (-0.27-0.15)	<0.001

MDD, major depressive disorder; FIPR, family income-to-poverty ratio; BMI, body mass index; WC, waist circumference; CVD, cardiovascular disease; CKD, chronic kidney disease; WBC, white blood cell; Neu, segmented neutrophil; Lym, lymphocyte; PLT, platelet; Hb, hemoglobin; ALT, alanine aminotransferase; AST, aspartate aminotransferase; ALP, alkaline phosphatase; GGT, gamma glutamyl transferase; Scr, serum creatinine, UA, uric acid; TC, total cholesterol; TG, triglyceride; LDL-C, low-density lipoprotein cholesterol; HDL-C, high-density lipoprotein cholesterol; FPG, fasting plasma glucose; HbA1c, glycated hemoglobin A1c; AIP, atherogenic index of plasma; PHQ-9, patient health questionnaire; Q1, quartile 1; Q2, quartile 2; Q3, quartile 3; Q4, quartile 4. MDD was defined as a PHQ-9 score greater than or equal to 10.

### 3.2 AIP cut-off value for MDD

As shown in **Table 2**, a statistically significant cut-off point (cut-off point=-0.42, *P*=0.013) in the relationship between AIP and MDD was identified using recursive partitioning analysis. An AIP of -0.42 is the threshold for the risk differentiation of MDD, and the OR value of AIP is close to 1. When the AIP is less than -0.42, it was negatively associated with the risk of MDD (OR=0.21, 95% *CI* 0.05~0.91). Conversely, when the AIP was greater than -0.42, it was positively related to the risk of MDD (OR=1.73, 95% *CI* 1.23~2.44).

**Table 2:** Cut-off point and segmentation effects of AIP in predicting depressive disorder.

Items	Outcomes
Linear effect	1.42 (1.04, 1.93)
Segmentation effect	
Cut-off point (K)	-0.42
< K segment effect	0.21 (0.05, 0.91)
> K segment effect	1.73 (1.23, 2.44)
Effect difference	8.17 (1.67, 40.04)
Logarithmic likelihood ratio test	0.013

### 3.3 The relationship between AIP and depression

Furthermore, multivariate linear and logistic regression model was performed to explore the correlation between AIP and PHQ-9 score and MDD, respectively (**Table 3**). In more detail, the results initially demonstrated a significant positive relationship between AIP and PHQ-9 scores in the crude model (β=1.15, 95% *CI* 0.84~1.45). In the fully adjusted model, a positive association between the AIP and PHQ-9 score was also observed (β=0.46, 95% *CI* 0.14~0.78), suggesting that each unit of increased AIP score was associated with 0.46 increased units of PHQ-9 score. We further converted the AIP from a continuous variable to a categorical variable to conduct the sensitivity analysis. Compared with the lowest AIP quartile, the PHQ-9 score increased with the higher AIP groups. The mean PHQ-9 score of the highest AIP quartile was 0.39 units higher than that of the lowest quartile (β=0.39, 95% *CI* 0.12~0.66, *P* for trend=0.004).

**Table 3:** The relationship between AIP and depression.

AIP	PHQ-9 score	MDD
	β (95% *CI*)	OR (95% *CI*)
Crude model (Model 1)		
Continuous	1.15 (0.84, 1.45)	2.21 (1.70, 2.87)
Categories		
Q1	0 (reference)	1 (reference)
Q2	0.21 (-0.06, 0.47)	1.17 (0.92, 1.50)
Q3	0.41 (0.15, 0.68)	1.37 (1.08, 1.74)
Q4	0.92 (0.66, 1.19)	1.85 (1.48, 2.32)
*P* for trend	<0.001	<0.001
Adjusted model (Model 2)		
Continuous	1.15 (0.84, 1.45)	2.12 (1.60, 2.81)
Categories		
Q1	0 (reference)	1 (reference)
Q2	0.21 (-0.04, 0.47)	1.12 (0.87, 1.44)
Q3	0.46 (0.20, 0.72)	1.35 (1.05, 1.73)
Q4	0.91 (0.64, 1.17)	1.78 (1.39, 2.26)
*P* for trend	<0.001	<0.001
Fully adjusted model (Model 3)		
Continuous	0.46 (0.14, 0.78)	1.42 (1.04, 1.93)
Categories		
Q1	0 (reference)	1 (reference)
Q2	0.06 (-0.19, 0.30)	1.03 (0.79, 1.33)
Q3	0.13 (-0.12, 0.39)	1.14 (0.87, 1.48)
Q4	0.39 (0.12, 0.66)	1.33 (1.02, 1.73)
*P* for trend	0.004	0.020

MDD, major depressive disorder; Q1, quartile 1; Q2, quartile 2; Q3, quartile 3; Q4, quartile 4; OR, odds ratio; 95% CI, 95% confidence interval. Model 1, no covariates were adjusted; Model 2, Adjusted for gender, age, race, education level, marital status, and FIPR; Model 3, Adjusted for gender, age, race, education level, marital status, FIPR, BMI, diabetes, hypertension, hyperlipidemia, CVD, CKD, FPG.

**Figure 2:**
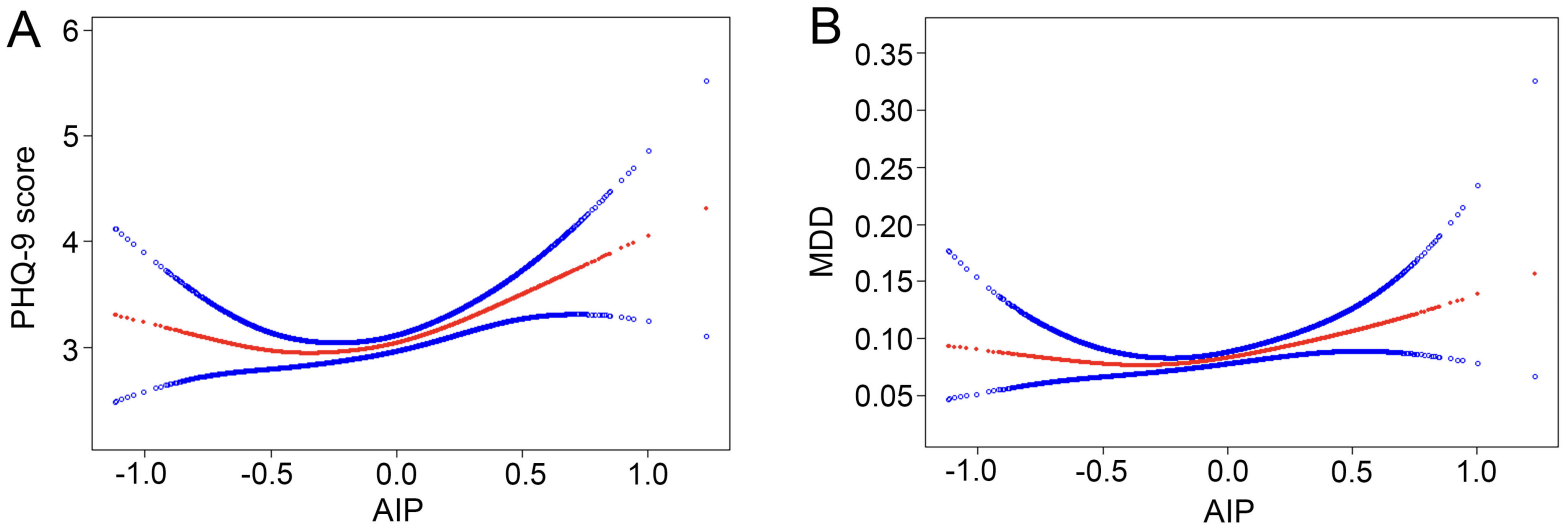
Nonlinear relationship between AIP and depression. The red line represents the smooth curve fit between variables, and the blue bands represent the 95% of confidence interval from the fit. **(A)** AIP and PHQ-9 score; **(B)** AIP and MDD. MDD, major depressive disorder.

For MDD, we also found a positive association between AIP and the increased likelihood of depressive disorder with statistical significance. After full adjustment in Model 3, subjects with a unit higher AIP had a 42% increased risk of MDD (OR=1.42, 95% *CI* 1.04~1.93). The association remained statistically significant after AIP was treated as quartiles (*P* for trend=0.020). Participants in the highest AIP quartile had a significantly 33% higher risk than those in the lowest AIP quartile (OR=1.33, 95% *CI* 1.02~1.73). Overall, the present findings implied that a higher AIP was associated with a higher PHQ-9 score and an increased risk of MDD. Smoothing plot analysis further validated the positive and non-linear association between AIP and PHQ-9 score and MDD (**Figure 2**).

### 3.4 Predictive power of AIP for MDD

**Figure 3:**
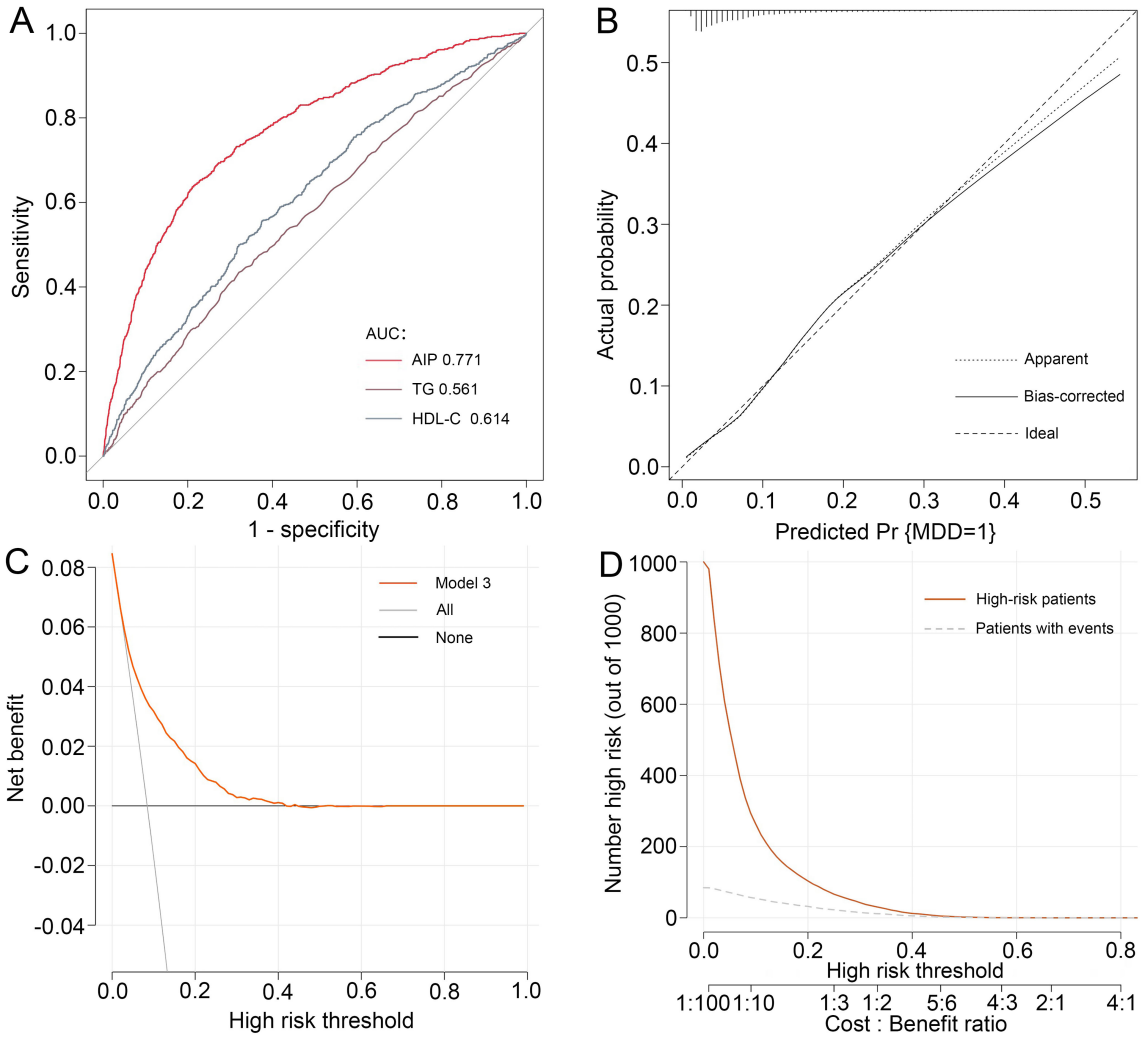
Performance evaluation of AIP for predicting MDD. After adjustment for confounding factors, predictive power of AIP for MDD was examined using **(A)** the area under the receiver operating characteristic curve (AUC), **(B)** calibration curve, **(C)** decision curve analysis (DCA), and **(D)** clinical impact curve (CIC) analysis. MDD, major depressive disorder.

To synthetically assess the performance of AIP in identifying MDD, the ROC curve, calibration curve, Hosmer-Lemeshow test, as well as the DCA and CIC were employed to process the analysis (**Figure 3**). After adjustment for confounding factors, ROC analysis showed that the AUC of AIP (AUC=0.771) was larger than that of TG (AUC=0.561) and HDL-C (AUC=0.614) alone, respectively. Moreover, the Hosmer-Lemeshow test (*χ^2^ =* 14.239, *P*=0.076) and calibration curve all suggested an excellent performance and goodness-of-fit of the multivariate model. DCA and CIC analysis were used to examine the clinical utility of the model, indicating a favorable overall net benefit and clinical impact within most reasonable threshold probability of the model.

### 3.5 Subgroup analysis

We conducted subgroup analyses and interaction tests stratified by age, gender, BMI, smoker, diabetes, hypertension, and CVD, and to evaluate whether the correlation between AIP and MDD was consistent in the general population and to determine any potentially different population settings (**Table 4**). The results of our study revealed that the association between AIP and PHQ-9 score and MDD was significantly different in the individuals with diabetes and hypertension (*P* for interaction<0.05). In the population with diabetes, the risk of MDD was 2.78-fold higher for every unit increase in AIP (OR=2.78, 95% *CI* 1.49~5.21). In hypertensive patients, individuals with a unit higher AIP had a 94% increased risk of MDD (OR=1.94, 95% *CI* 1.30~2.91).

**Table 4:** Subgroup analysis of the association between AIP and depression.

Subgroups	PHQ-9 score			MDD	
	β (95% *CI*)		*P* for interaction	OR (95% *CI*)	*P* for interaction
Age (years)		0.983			0.771
<65	0.48 (0.09, 0.86)			1.38 (0.97, 1.96)	
≥65	0.47 (-0.08, 1.02)			1.52 (0.85, 2.73)	
Gender		0.836			0.907
Male	0.45 (0.01, 0.89)			1.47 (0.93, 2.33)	
Female	0.51 (0.06, 0.97)			1.42 (0.95, 2.11)	
BMI (kg/m^2^)		0.407			0.764
<25	0.36 (-0.26, 0.98)			1.40 (0.75, 2.59)	
≥25	0.66 (0.29, 1.03)			1.55 (1.09, 2.21)	
Smoker		0.083			0.132
Yes	0.31 (-0.12, 0.75)			1.37 (0.94, 2.00)	
No	-0.24 (-0.69, 0.22)			0.85 (0.52, 1.40)	
Diabetes		0.030			0.014
Yes	1.19 (0.46, 1.92)			2.78 (1.49, 5.21)	
No	0.32 (-0.03, 0.67)			1.16 (0.82, 1.64)	
Hypertension		0.030			0.010
Yes	0.81 (0.36, 1.26)			1.94 (1.30, 2.91)	
No	0.11 (-0.34, 0.56)			0.86 (0.53, 1.40)	
CVD		0.640			0.334
Yes	0.57 (0.02, 1.13)			1.23 (0.81, 1.86)	
No	0.42 (0.05, 0.79)			1.67 (1.06, 2.63)	

MDD, major depressive disorder; BMI, body mass index; CVD, cardiovascular disease; OR, odds ratio; 95% CI, 95% confidence interval.

## 4 Discussion

This population-based study of 7,951 US adults form NHANES 2005-2018 explored the relationship between AIP and the incidence of depressive disorder. Notably, the results confirmed that the elevated AIP levels were linked to a higher prevalence of MDD, and this relationship also remained significant even after accounting for all confounding factors, with the highest AIP quartile raising the risk by 33% in the population. Meanwhile, predictive power test indicated an excellent performance and a favorable overall net benefit of the multivariate model. In subgroup analyses and interaction tests, our findings implied that AIP was positively correlated with MDD and a significant dependence of diabetes and hypertension on this correlation was further observed, indicating that elevated levels of AIP may lead to higher PHQ-9 scores and increased the risk of developing MDD, particularly in diabetic and hypertensive populations.

Recently, the metabolic profile leading to depression has attracted much attention (20). AIP serves as a reliable indicator of the atherogenic potential of lipoproteins, particularly the small, dense, and cholesterol-rich particles that play a significant role in the pathogenesis of atherosclerosis (21). Atherosclerosis, as a primary pathological mechanism, significantly accelerates the initiation and progression of multiple cardiovascular diseases (22). Of note, a clear correlation between the presence of CVD and the incident depressive disorder has been well established (5, 23, 24). Moreover, previous evidence (25, 26) implicated that chronic inflammatory states act as a bridge linking depression and atherosclerosis. Studies (27–29) have shown that depression is often accompanied by elevated levels of pro-inflammatory markers such as C-reactive protein and interleukin-6, which not only lead to the onset and progression of depression but also significantly increase the risk of CVD. AIP, which is considered to be involved in both atherosclerosis and inflammation, is expected to be a key link between the two health problems (30, 31). Thus, further study of AIP as a predictor of the presence of depressive disorder may markedly contribute to enhancing our understanding of the intricate biological processes linking mental disorders with cardiovascular health.

Consistent with previous study (32), our current study demonstrated a significant association between elevated AIP levels and a greater prevalence of MDD, even after adjusting for potential confounding factors. This revealed that a more severe disturbance in metabolic profile is related to a higher incidence of MDD, and this correlation between AIP levels and MDD prevalence remained consistent across most subgroups. Notably, individuals with diabetes or hypertension showed a robust association between AIP levels and MDD prevalence, unlike their non-diabetic or non-hypertensive counterparts. Diabetes has been shown to be an important risk factor for MDD (33). Recent research (34) shows that depressive disorder is associated with a higher risk of diabetes, and comorbidity of the two worsens long-term survival. Consequently, blood glucose management and prevention of diabetes should be emphasized in depressed patients, and diabetic patients should also be routinely screened for depression risk considering the impact of comorbid diabetes and depressive disorder. Similarly, hypertension also plays an important role in the development of depressive disorder (35). Results from a cohort (36) comprising 8,677 participants suggested depressive disorder is associated with the risk of cardiovascular death in hypertensive patients, and the more severe the depressive symptoms, the higher the risk of cardiovascular death. Furthermore, a significant bidirectional phenotypic association between depression and hypertension was further recorded in an observational analysis of more than 400,000 participants (37). Most importantly, a multi-center, parallel, cluster-randomized, controlled, implementation trial (38) confirmed that the addition of depression care to chronic medical care exerted a more positive effect on improving mental health outcomes for individuals with hypertension and diabetes. Surprisingly, AIP levels were positively correlated with MDD in individuals younger than 65 years of age or with CVD, but the risk of MDD in the subgroups was almost consistent from the perspective of the two general populations. The difference may derive from the multifaceted physiological and psychological effects of age and CVD history, thus masking a clear link between depressive disorder and the two. Elderly people and those with CVD history may be more aware of risk factors for depressive disorder and tend to adopt healthier lifestyle habits that positively alter lipid metabolism and cardiovascular health, partially offsetting the impact of age and comorbidities on the association of depression. However, residual physiological effects of advanced age and CVD on depression may still remain, and these potential effects can indirectly influence the association of AIP with depression (39, 40). Our findings highlight the importance of having to consider numerous factors when addressing depressive disorder, including multiple lipid factors and comorbidities.

Several reasons may explain the strong association between elevated AIP levels and increased incident depressive disorder. First, previous study (41) suggested that increased lipids are often accompanied by an increase in chronic inflammation, which may affect the immune system and emotional regulation, thereby leading to depressive disorders. Dysregulated neuroinflammatory status might underlie the antidepressive-like behaviors (42). Substantial evidences have demonstrated that monophosphoryl lipid A, through immune stimulation, may prevent stress-induced depression-like behaviors in mice by preventing neuroinflammatory responses (43, 44). Second, metabolic disorders often cause neuroendocrine disorders, such as insulin resistance, increased glucocorticoid secretion, and disturbed glucocorticoid sensitivity associated with systemic cortisol activity, which may interfere with neurogenesis and neuroplasticity (45–47). Moreover, oxidative stress response and endothelial dysfunction, widely recognized as central to the development of CVD, may also occupy a key position in the pathophysiology of depression (48, 49). Given the strong link between AIP and MDD, it is critical to reduce the likelihood of MDD in clinical settings by thoroughly evaluating individuals with elevated AIP levels and implementing effective interventions to mitigate risk factors.

This study has several strengths. Firstly, the data used in this study came from NHANES, a nationwide population-based sample database that followed a standard protocol. All analyses were conducted with consideration of appropriate NHANES sampling weights to make the study samples more representative. Secondly, after adjusting for confounder factors, the present study simultaneously evaluated nonlinear associations between AIP and PHQ-9 score and MDD, calculated the cut-off point, and determined segmented effects between variables to provide more robust evidence. However, several limitations of the present study must be acknowledged. Firstly, due to the cross-sectional study design, we cannot obtain a clear causal relationship between AIP and the incidence of depression. Besides, it is worth noting that the AIP measurement was assessed in a timely manner in this study, and the results may not reflect the patients’ long-term situation. the diagnosis of MDD in this study was obtained through self-reporting by participants. Secondly, although certain measures were taken during data collection to avoid systematic errors, there may still remain information bias. Thirdly, we converted AIP into a categorical variable for further analysis, which may lead to a lack of confidence in the evidence due to the reduced sample size in each group. Therefore, future studies can be conducted by expanding the sample size or focusing on a specific population to explore the potential mechanisms of lipid management and depressive disorder.

## 5 Conclusion

The current investigation showed a positive and non-linear correlation between elevated AIP levels and higher PHQ-9 scores and a greater risk of MDD. Our findings emphasized the necessity of lipid management in identifying patients at risk of depressive disorder and suggested that AIP may serve as a valuable indicator for evaluating MDD risk in individuals, particularly those with diabetes and hypertension. Most importantly, the present study suggested a potential method of evaluating lipid levels and optimizing the risk stratification of depression.

## Data Availability

The original contributions presented in the study are included in the article/supplementary material. Further inquiries can be directed to the corresponding author.
